# Alginate Oligosaccharide DP5 Exhibits Antitumor Effects in Osteosarcoma Patients following Surgery

**DOI:** 10.3389/fphar.2017.00623

**Published:** 2017-09-12

**Authors:** Jiayu Chen, Yu Hu, Lirong Zhang, Yingjian Wang, Shichao Wang, Yanzi Zhang, Haiyan Guo, Degang Ji, Yingtao Wang

**Affiliations:** ^1^Department of Basic Medicine, Medical School, Shaoxing University Shaoxing, China; ^2^Department of Pathology, China-Japan Union Hospital of Jilin University Changchun, China; ^3^Department of Gynecology and Obstetrics, China-Japan Union Hospital of Jilin University Changchun, China; ^4^Wenzhou Medical College Wenzhou, China; ^5^Department of Pathology, Tumor Hospital of Jilin Province Changchun, China; ^6^Department of Hepatobiliary Pancreatic Surgery, China-Japan Union Hospital of Jilin University Changchun, China; ^7^Pharmacy Department, China-Japan Union Hospital of Jilin University Changchun, China

**Keywords:** alginate oligosaccharide, local recurrence, osteosarcoma, tumor volume, antioxidant, anti-inflammatory

## Abstract

Osteosarcoma is a malignant musculoskeletal tumor that has high-rate morbidity and mortality worldwide. Alginate oligosaccharide (AOS), a natural product, has antitumor activities and may have therapeutic effects in osteosarcoma, the molecular mechanisms of which remain unclear. AOS was prepared from alginate sodium using alginate lyase. The fractions of AOS were further isolated by size-exclusion chromatography and verified by electrospray ionization mass spectrometry (ESI-MS). Osteosarcoma patients were enrolled in the study and assigned into two groups: AOS (AG, oral administration of 10-mg AOS daily) and control groups (CG, placebo). Preoperative and postoperative clinical data were investigated and analyzed. Four different degrees of polymerizations (DPs) were isolated and denominated as DP2, DP3, DP4, and DP5. Among these polymers, only DP5 showed antitumor functions on osteosarcoma cells. Before surgery and the outcome of primary end point after surgery, no significant differences were observed for clinical data and tumor size between the AG and CG groups (*P* > 0.05). After 2-year therapy, the mean tumor volume was 214.6 ± 145.7 c.c. in AG and 467.2 ± 225.3 c.c in CG (*P* < 0.01). The rate of local recurrence was 44.9 and 68.7% in AG and CG, respectively (*P* < 0.01). AOS treatment resulted in the increase in serum levels of SOD, GSH, HDL-C, and reduction in the levels of interleukin-1 (IL-1) beta and IL-6; the ratios of AST/ALT; and triglycerides, total cholesterol (TC), low-density lipoprotein cholesterol LDL-C, and malondialdehyde (MDA) (*P* < 0.05). AOS reduces osteosarcoma progression, which is associated with improvement in antioxidant and anti-inflammatory capacities of patients, and may be used as a potential drug for osteosarcoma therapy.

## Introduction

Osteosarcoma (OS) is the most common type of bone cancer and affects children and adolescents worldwide (Angulo et al., 2017). OS has high morbidity and mortality. Moreover, the use of chemotherapy (cisplatin; Chen, 2017; Sun et al., 2017), doxorubicin (Hattinger et al., 2017; Zhang et al., 2017), and methotrexate (Valle et al., 2016; Ray et al., 2017) results in marginal improvement in survival rate even after surgery. The most serious problem is that survival rates have changed little over the past decades because no new drug is available. Therefore, exploring potential drugs with few side effects from plants is needed.

Oligosaccharide from dietary sources have been considered to inhibit cancer growth and impede cancer development (Kapoor and Dharmesh, 2017). It has good biocompatibility, is non-toxic and can be obtained cheaply. Hyaluronan oligosaccharides have been reported to inhibit tumorigenicity of murine osteosarcoma cell, LM-8, and human osteoblastic osteosarcoma cell, MG-63, via disruption of receptor–hyaluronan interaction (Hosono et al., 2007). Oral administration of chitosan oligosaccharides (COS) resulted in an ~60% reduction of tumor size and tumor numbers/sectioning for colorectal cancer (CRC) in a mouse model (Mattaveewong et al., 2016). Konjac glucomannan and inulin oligosaccharide can inhibit the development of colitis-associated colon carcinogenesis (Wu et al., 2016).

Alginate oligosaccharide (AOS), as a kind of non-immunogenic, non-toxic and biodegradable polymer, has been reported to have antioxidant, anti-inflammatory and anti-endoplasmic reticulum stress effects (Guo et al., 2016). AOS is prepared from alginate sodium, a major component of sea algae, which are the most abundant plant resource in the ocean. Metastasis is the main reason for causing cancer-related deaths. Transforming growth factor beta (TGF)-beta promotes cancer development and metastasis by activating epithelial-mesenchymal transition (EMT) (Feng et al., 2017; Mo et al., 2017). Marine-derived oligosaccharides have been found to prevent the progression of metastatic malignancies by inhibiting TGF-mediated EMT (Zhou et al., 2016).

AOS has been known to have anti-bacterial and anti-biofilm properties. It also possesses antibiotic activity against multi-drug resistant bacterial pathogens. In the rational drug design for chemotherapy, AOS shows promising application in tumor therapy (Yang et al., 2016b). Alginate consists of the 1,4-linked epimers α-L-guluronate (G) and β-D-mannuronate (M) with polyguluronate (PG), polymannuronate (PM), or alternating sequences of mannuronate and guluronate (polyMG). AOS can be prepared with various degrees of PG and PM from alginate by using alginate lyase. Earlier results suggest that the chemical properties of AOS are correlated with antitumor activity (Fujihara and Nagumo, 1992). Depolymerized guluronate and mannuronate oligomers can induce cytotoxic cytokines in human cells. Comparatively, guluronate oligomers have higher cytotoxicity than mannuronate oligomers (Iwamoto et al., 2003). However, the effects of AOS on OS are seldom reported. All the information suggests that AOS may have some beneficial effects on inhibiting OS progression although the effects of AOS on OS are still unclear and related molecular mechanisms remain unknown. Therefore, we investigated the effects of AOS on OS by using its specific polymers of AOS. The inhibitory functions on OS were confirmed by investigating the tumor volume, local recurrence, and antioxidant and anti-inflammatory characteristics in the patients after surgeries.

## Materials and methods

### AOS prepared from alginate sodium

The AOS with α-L-guluronate units and β-D-mannuronate units was bought from Qingdao Qingya Chemical Co., Ltd. (Qingdao, China). According to an earlier report (Han et al., 2015), AOS was prepared from alginate sodium using alginate lyase, which depolymerizes alginate sodium of brown algae. The alginate lyase, from *Agarivorans* sp. L11, was cloned and expressed in *Escherichia coli* according to a previous report (Li et al., 2015). The recombinant enzyme exhibited an activity of 1,370 U/mg in liquid at 39°C. Briefly, 1 kg sodium alginate was depolymerized in 100 L tap water with 10 mg of alginate lyase at 39°C for 2 h. The lyase was denatured at 110°C for 10 min. The amounts of unsaturated saccharides were estimated by measuring the absorbance at 234 nm in a UV-2100PC UV-VIS spectrophotometer (Shimadzu, Kyoto, Japan).

### Isolation and purification of AOS

AOS was purified and dried by the evaporation of water and ammonium acetate at 20 mbar and 65°C, after which AOS was obtained in sodium salt form. The depolymerization of AOS was further isolated and purified by size-exclusion chromatography. A total of 10 g of powder was dissolved in 20 mL water and loaded to a 6 × 60 cm Superdex 75 prep grade column (GE Healthcare, Aurora, OH, USA), which was equilibrated with the buffer including 50 mm sodium phosphate, pH 7.5, and 300 mM NaCl. The column was eluted with the same buffer at a linear flow rate of 0.5 cm/min and the fractions were measured by ultraviolet absorption at 280 nM. The peak fractions were collected and the component was characterized by electrospray ionization mass spectrometry (ESI-MS).

### Characterization of purified AOS

The degree of polymerization (DP) was further determined by ESI-MS. The hydrolysate was passed through a carbograph column to remove salt, and then concentrated, dried and dissolved in 1 mL methanol. A total of 2 μL samples were injected to a LTQ XL mass spectrometer (Thermo Finnigan, Austin, TX, USA). AOS was detected in a positive-ion mode: ion source, 5 kV; capillary temperature, 280–310°C; Tube lens, 260 V; and sheath gas, 35 arbitrary units (AU); The mass spectrometer was set over a range of *m*/*z* 0–1,500.

### Cell culture

To understand the different functions of specific polymers of AOS, cell culture was performed by adding different DPs of AOS. Human bone cancer cell MG-63 was purchased from the cell bank of CAS (Shanghai, China), and cultured in DMEM at 37°C with 5% CO_2_. After 3-day culture, the cells were treated with trypsin and pooled. The cells were washed thrice with fresh DMEM. The cell concentration was adjusted to 1 × 10^5^ cells/mL; 200 μL cells were transferred to each well in a 96-well plate, treated with different AOS DPs, and further cultured for 3 days under the same situation. Cell growth was recorded by an xCELLigence system (Roche, Indianapolis, IN, USA).

### Participants

This study was carried out in accordance with the recommendations of Human Research Guidelines of the Ethical Committee of China-Japan Union Hospital of Jilin University with written informed consent from all subjects. All subjects gave written informed consent in accordance with the Declaration of Helsinki. The protocol was approved by the committee of China-Japan Union Hospital of Jilin University. From June 1, 2012 to April 1, 2015, the OS patients, who underwent surgical removal of tumor, were recruited in the study.

### Inclusion criteria

Inclusion criteria included patients with OS characteristics. All patients needed to be treated with the combination of chemotherapy and surgery. Chemotherapy therapy consisted of two cycles of cisplatin, doxorubicin, and methotrexate for 2 months. Bevacizumab was administered for 3 days before the chemotherapy and subsequently on the first day of weeks three and five. All patients underwent similar chemotherapy. They came from similar economic and cultural background.

### Exclusion criteria

The following exclusion criteria were used: the patients underwent amputation surgeries; the patients were from other hospitals; the OS patients were died within 4 months postoperatively; the patients had no complete clinical data; the patients used nutritional supplements, such as calcium and omega-3 fatty acids; and females who developed OS during pregnancy.

### Patient grouping

After screening inclusion and exclusion criteria, 108 OS patients were selected to join the present experiment. Fifty-four patients received 10 mg of AOS orally once daily and assigned as the AOS group (AG). Fifty-four patients received 10 mg of placebo orally once daily and assigned as the control group (CG). All baseline characteristics were normalized between the two groups. Their preoperative and postoperative clinical data were recorded. Histologically, OS is classified into G1, G2, G3, and G4 (Enneking et al., 1980). OS sizes were measured by conventional magnetic resonance imaging (MRI) and calculated using the formula: ([π/6] × length × width × depth). The mean follow-up period was 24 months. OS samples were stained with hematoxylin and eosin (H&E) (Yang et al., 2016a).

### Biochemical analysis

AST and ALT were measured by an automated clinical chemistry analyzer (Indianapolis, IA, USA). The activity of SOD was assayed according to the method reported by Misra and Fridovich (1977). GSH was assayed via the reaction with 5,5′-dithiobis(2-nitrobenzoic acid) (DTNB) and measured at 412 nm. Evidence indicates that the risk of the occurrence of many tumors is associated with blood lipid profile [high-level total cholesterol (TC), glycerol, and low-density lipoprotein cholesterol (LDL-C), and high-density lipoprotein cholesterol (HDL-C) levels] (Hattinger et al., 2017; Ray et al., 2017). Thus, the lipid profile, which may be related to OS risk, was measured, including total triglycerides (TGs), TC, HDL-C, and LDL-C using a biochemical analyzer (Dimension, Schererville, IN, USA). Malondialdehyde (MDA) was assayed using an MDA kit manufactured from Sobioda (Grenoble, France).

### Measurement of inflammatory cytokines

The pathogenesis of OS is related to interleukin-1 (IL-1) beta (He et al., 2014) and IL-6 (Qi et al., 2016). A total of 5 mL of blood samples were taken from all OS patients. The levels of IL-1 beta and IL-6 were measured by using the kits from R&D Systems (Minneapolis, MN, USA). Serum TNF-α was measured by the kit from Abcam (Cat. no. ab181421).

### Statistical analysis

Student's *t-*test was used to compare the significant differences for variable parameters in two groups. Chi-square test was performed to investigate the significant differences in the numbers of two groups. Statistical analysis was performed by using the SPSS 20.0 (SPSS Inc., Chicago, IL, USA). Statistically significant difference was observed at *P* < 0.05.

## Results

### Characterization of AOS

**Figure 1:**
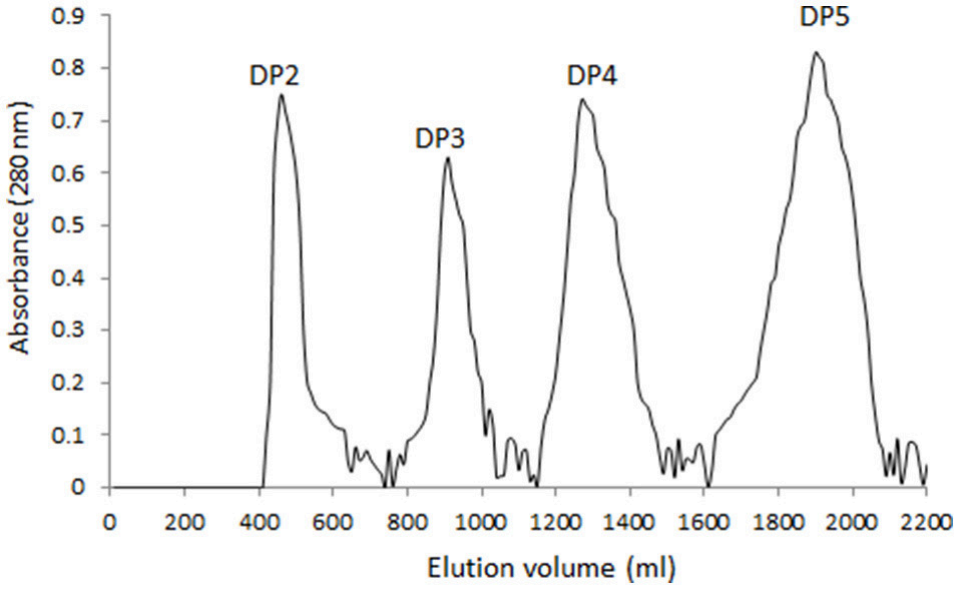
Gel chromatograms of the fractions from digested AOS. A 6 × 60 cm Superdex 75 prep grade column was eluted by 50 mm sodium phosphate buffer, pH 7.5 and 300 mM at a linear flow rate of 0.5 cm/min. Each fraction was measured at 280 nm via ultraviolet absorption.

**Figure 2:**
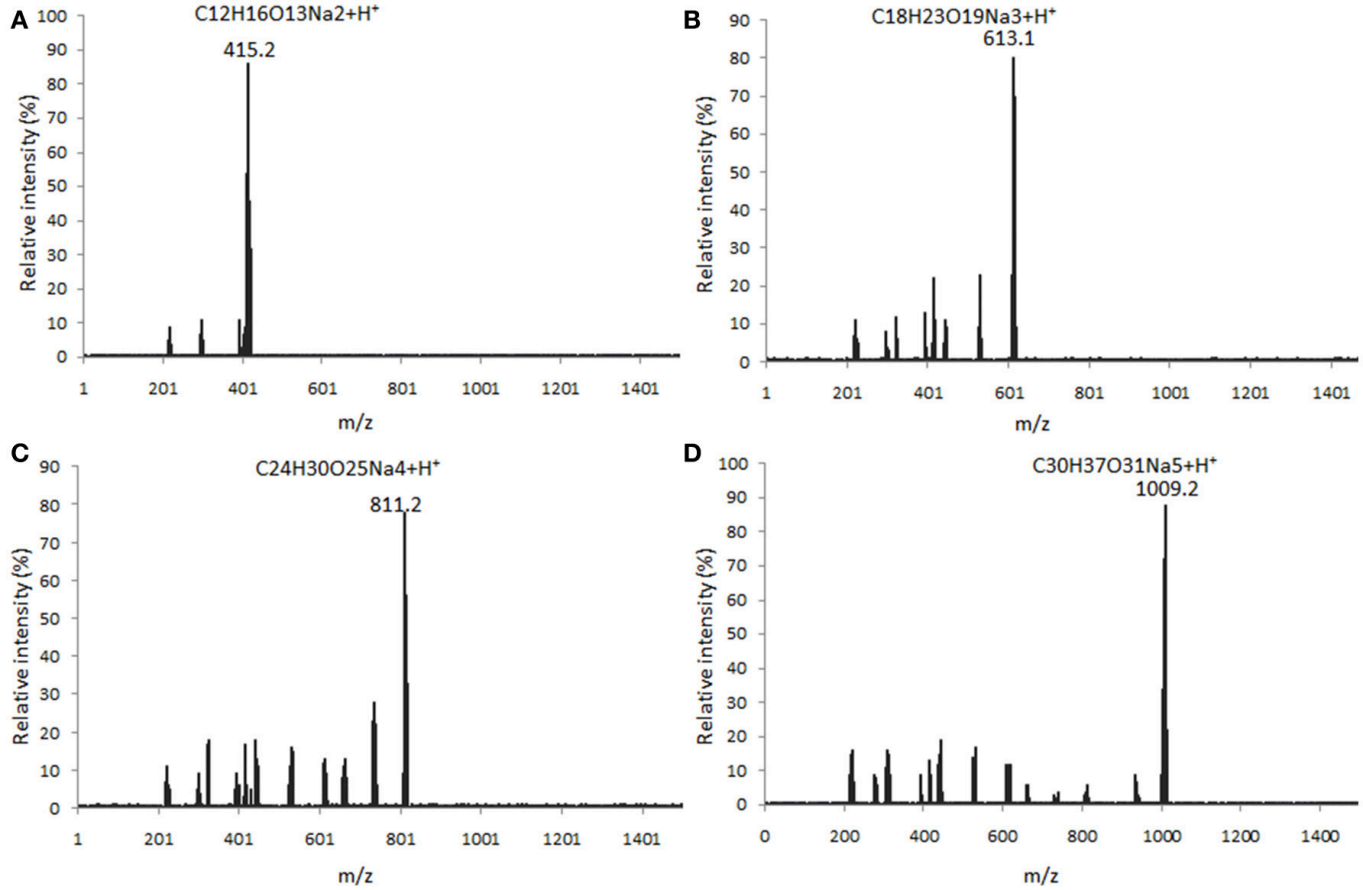
ESI MASS spectrometry analysis of the polymer degrees of digested AOS from alginate sodium under the conditions that produced mass spectra with M + H^+^. **(A)** mass spectra were visualized following the separation of DP2 ([M + H]^+^ = 415 Da); **(B)** mass spectra were visualized following the separation of DP3 ([M + H]^+^ = 613 Da); **(C)** mass spectra were visualized following the separation of DP4 ([M + H]^+^ = 811 Da); and **(D)** mass spectra were visualized following the separation of DP5 ([M + H]^+^ = 1,009 Da).

Four main components (DP2, DP3, DP4, and DP5) were isolated from alginate sodium using enzyme digestion (Figure 1). The isolated fractions of AOS were further confirmed by ESI-MS under the conditions that produced mass spectra with [M + H]^+^. The predicted masses for DP2 (C12H16O13Na2, Figure 2A), DP3 (C18H23O19Na3, Figure 2B), DP4 (C24H30O25Na4, Figure 2C), and DP5 (C30H37O31Na5, Figure 2D) were 414, 612, 810, and 1,009 Da, respectively.

### DP5 of AOS inhibits the growth of MG-63 cells

**Figure 3:**
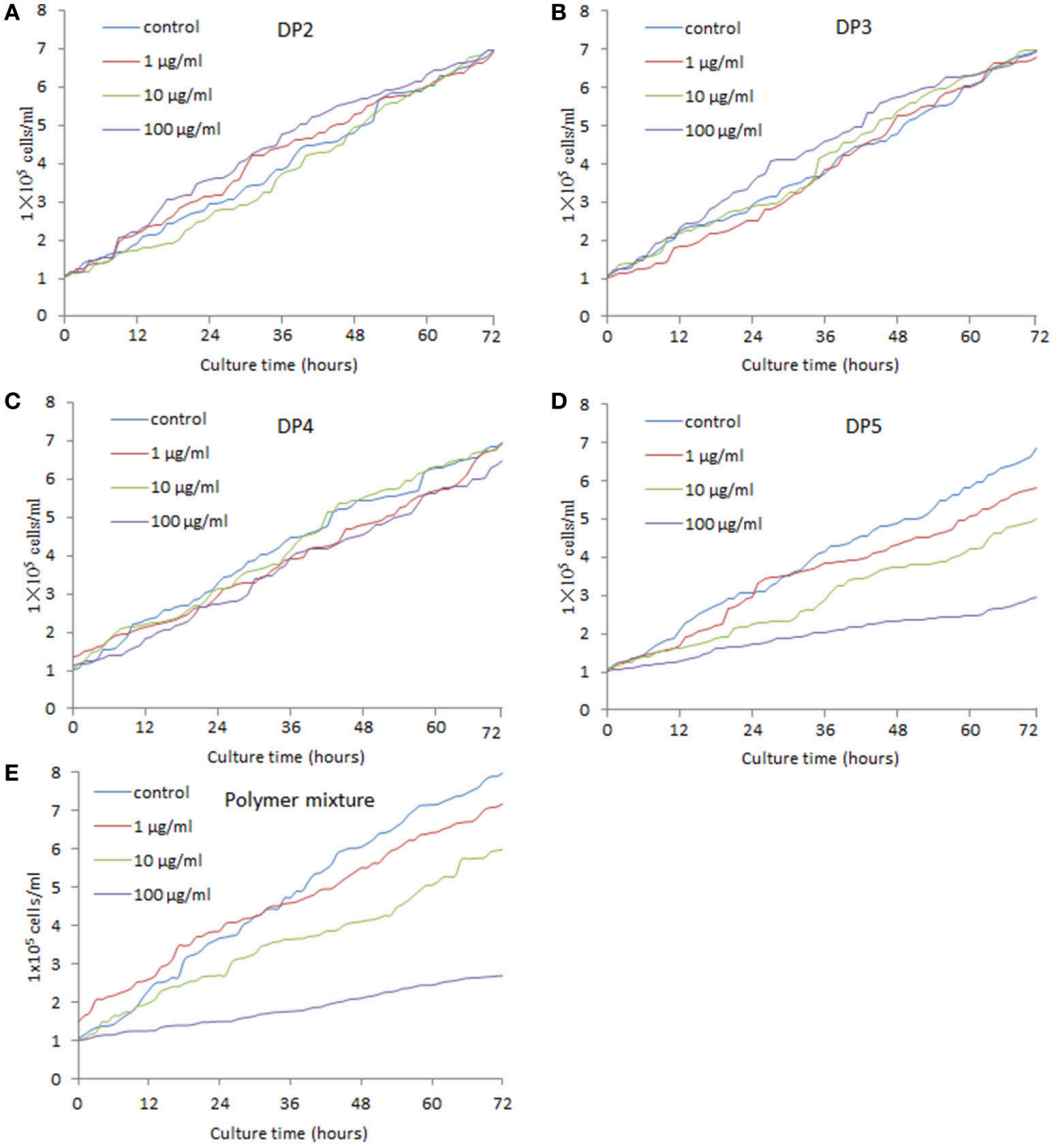
Real-time analysis for the effects of AOS on the growth of MG-63 osteosarcoma cells. **(A)** The effects of AOS DP2 on the growth of MG-63 cells. **(B)** The effects of AOS DP3 on the growth of MG-63 cells. **(C)** The effects of AOS DP4 on the growth of MG-63 cells. **(D)** The effects of AOS DP5 on the growth of MG-63 cells. **(E)** The effects of polymer mixture on the growth of MG-63 cells.

Figure 3A showed that DP2 of AOS did not inhibit the growth of MG-63 cells with the increase of its concentration. Comparatively, DP3 and DP4 (Figures 3B,C) could not change the growth rate of MG-63 cells either, even with the increase of their concentrations. By contrast, DP5 of AOS inhibited the growth of MG-63 cells with the increase of its concentration (Figure 3D). The polymer mixture of AOS can lower the function of DP5 when compared with only DP5 (Figures 3D,E). However, no statistically significant difference was observed between DP5 and mixture (*P* > 0.05). The results suggest that AOS inhibits the growth of osteosarcoma cells via high-degree polymers, such as DP5.

### Baseline characteristics of participants

A total of 88 patients were proven to have osteosarcomas and underwent surgical removal of tumors. The average ages of OS patients were 24.8 ± 12.6 and 23.5 ± 12.9 years in the AG and CG groups, respectively. The most common sites of OS were located at the distal end of femur. Average follow-up period was 2 years (ranging from 5 to 76 months). For other parameters, including OS grades, no statistically significant difference was observed between AG and CG (Table 1, *P* > 0.05).

**Table 1:** Baseline characters of osteosarcoma patients.

	**AOS group (n = 44)**	**Control group (n = 44)**	***P*-values**
Age, years	24.8 ± 12.6	23.5 ± 12.9	0.26
Gender, male/female	26/18	25/19	0.83
Smoking, n (%)	18(40.9)	20(45.5)	0.67
Alcoholic intake, n (%)	16(36.4)	17(38.6)	0.83
Body mass index, kg/m^2^	25.3 ± 6.4	24.8 ± 7.1	0.18
**OSTEOSARCOMA GRADES**			
G1, n (%)	8(18.2)	7(15.9)	0.78
G2, n (%)	9(20.5)	10(22.7)	0.80
G3, n (%)	19(43.2)	21(47.7)	0.67
G4, n (%)	8(18.2)	7(15.9)	0.78
Chemotherapy, n (%)	35(79.5)	33(75)	0.61
Resection length, cm	13.1 ± 5.7	12.8 ± 6.1	0.29
Stem diameter, cm	12.7 ± 3.2	11.8 ± 4.2	0.16
**SITE**			
Femur, n (%)	31(70.5)	30(68.2)	0.82
Tibia, n (%)	12(27.3)	11(25)	0.81
Others, n (%)	4(9.1)	7(15.9)	0.33
**HISTOLOGIC TYPE**			
Osteoblastic, n (%)	20(45.5)	18(40.9)	0.67
Chondroblastic, n (%)	14(31.8)	17(38.6)	0.51
Fribroblastic, n (%)	9(20.5)	8(18.2)	0.79
Others, n (%)	4(9.1)	5(11.4)	1.00
**DIFFERENTIATION STATUS**			
High, n (%)	31(70.5)	33(75)	0.63
Low, n (%)	16(36.4)	15(34.1)	0.82
Pulmonary metastasis, n (%)	4(9.1)	5(11.4)	1.00

**Figure 4:**
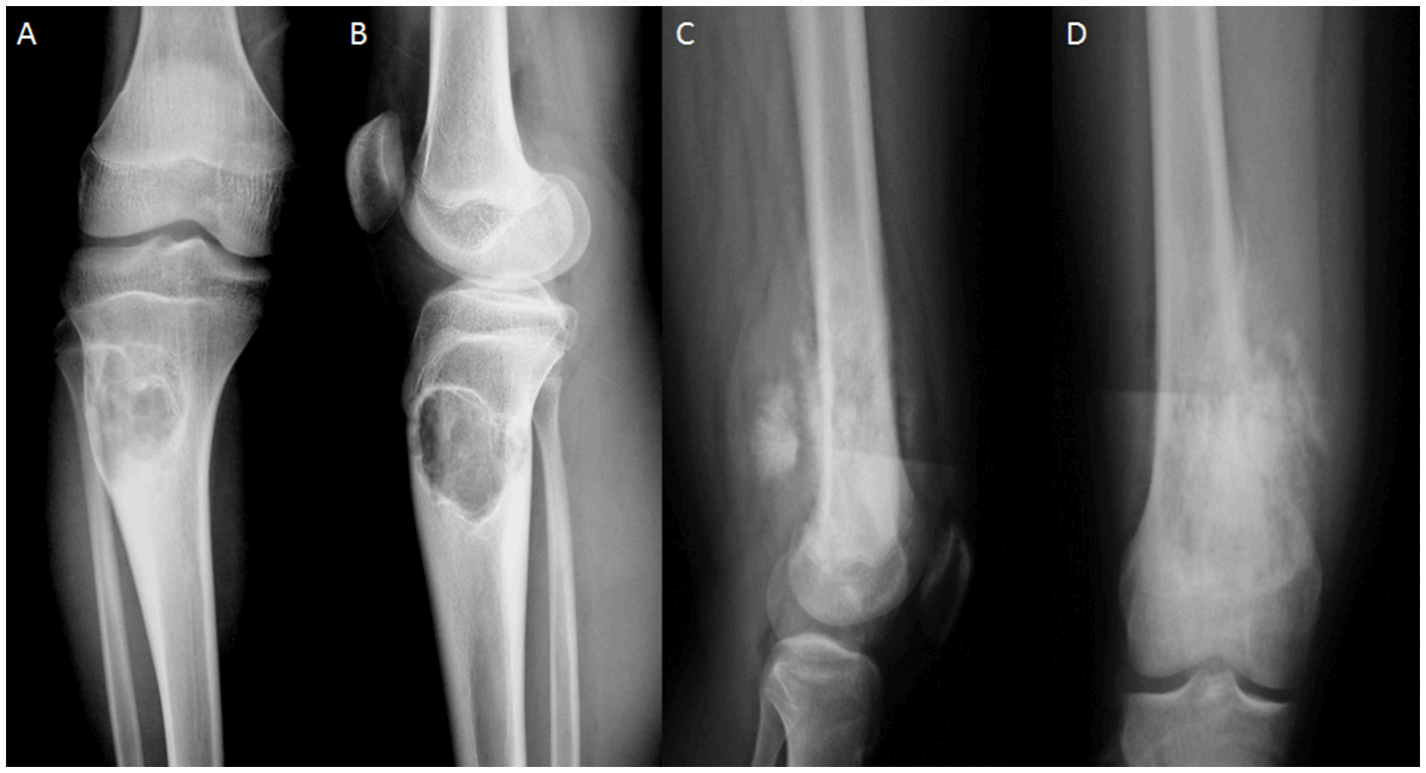
MRI images of OS at different stages. **(A)** OS at G1 grade. **(B)** OS at G2 grade. **(C)** OS at G3 grade. **(D)** OS at G4 grade.

Figure 4 shows the MRI images of OS at different stages. The size of OS was smaller at G1 (Figure 4A) than G2 (Figure 4B). With OS development, bone intensity was reduced greatly at G3 (Figure 4C) and G4 (Figure 4D) when compared with the bone tissues at G1 and G2 stages.

**Figure 5:**
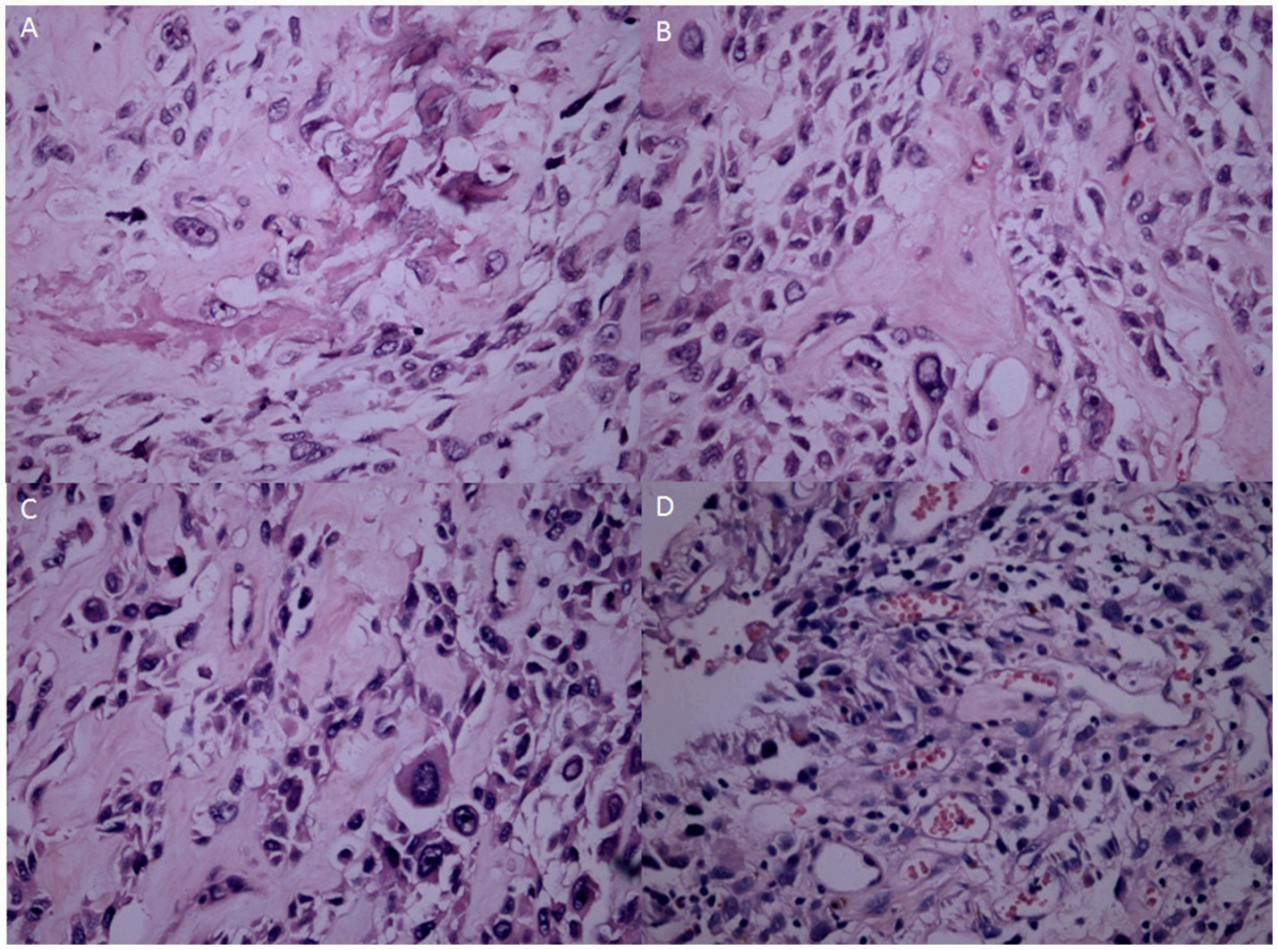
H&E staining analysis of OS samples at different stages. **(A)** The OS consists of an atypical round to cell proliferation with osteoid deposition at G1 stage (H&E stain ×200). **(B)** OS cells shape variable with chromatic nuclei and mitosis fields at G2 stages. **(C)** Spindle cell neoplasm has high cellularity, abnormal mitotic characters and atypical nuclear at G3 stage. **(D)** The OS forms a highly-cancerous and high-mortality bone tumor at G4 stage.

**Figure 6:**
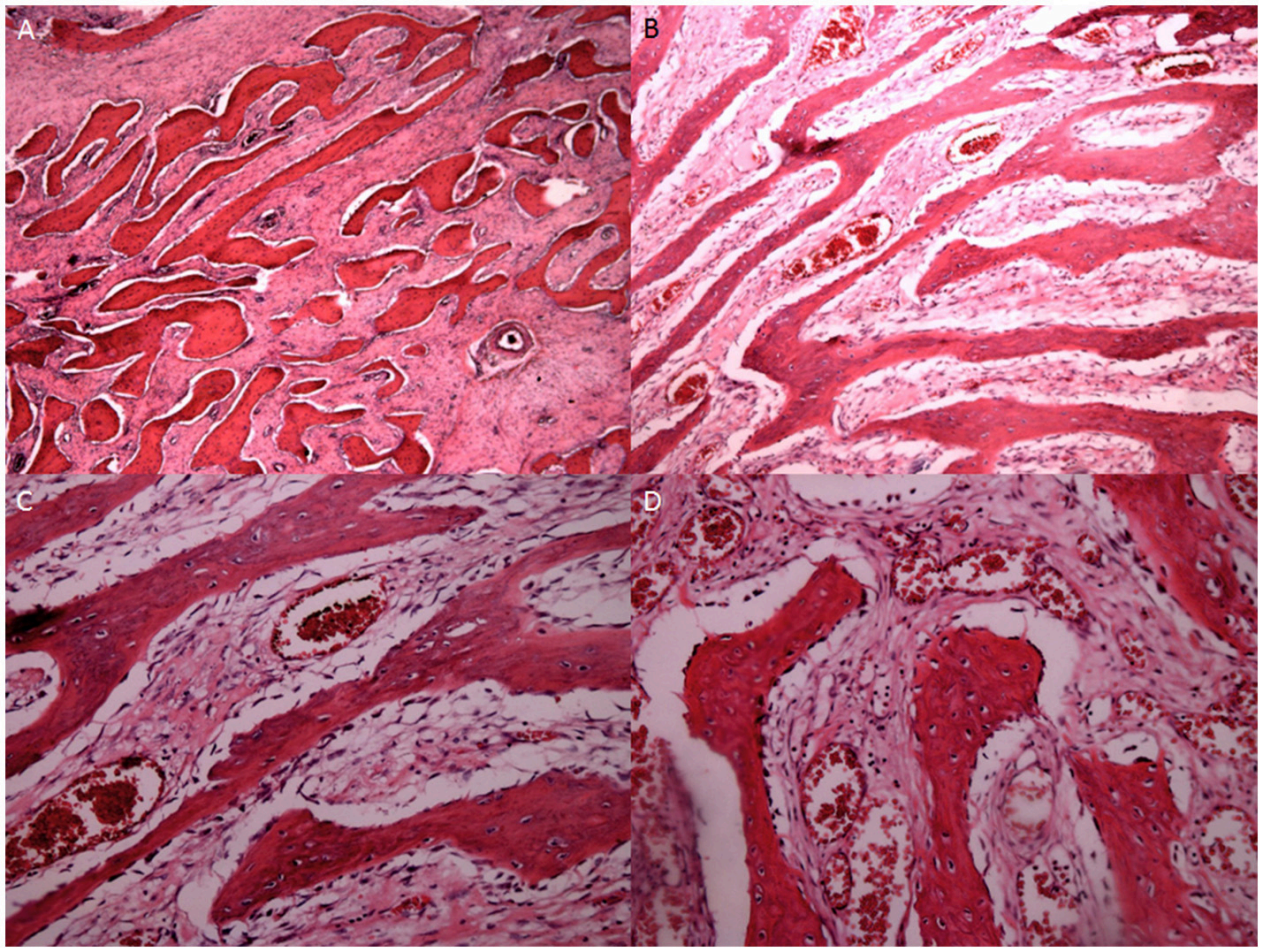
Histopathological examination of intraosseous vascular granulation tissue at different stages of OS (H&E stain ×200). **(A)** Internal hemorrhage were observed within the bone tissues at G1 stage. **(B)** Granulation tissue and responsive hyperosteogeny increased at G2 stage. **(C)** Granulation tissue and responsive hyperosteogeny increased further at G3 stage. **(D)** Giant granulation tissue and responsive hyperosteogeny at G4 stage.

H&E showed the OS tissues at different stages. At G1, the OS showed atypical cell proliferation with osteoid deposition and amorphous pattern (Figure 5A). At G2, OS cells were variable in shape with chromatic nuclei and mitosis (Figure 5B). At G3, spindle cell neoplasms had high cellularity, abnormal mitotic characters, and atypical nuclear characteristics (Figure 5C). At G4, the OS formed a high-mortality bone tumor (Figure 5D). Histopathological examination showed that intraosseous vascular granulation tissue increased with the OS development. At G1 stage, internal hemorrhage was observed within the bone tissues (Figure 6A). At G2 stage, granulation tissue and responsive hyperosteogeny increased when compared with the tissues at G1 (Figure 6B). At G3, granulation tissues and responsive hyperosteogeny increased further (Figure 6C). At G4, large granulation tissues and responsive hyperosteogeny were observed (Figure 6D).

### Long-term consumption of AOS reduces osteosarcoma size

No statistical significance was observed for tumor volume between the AG and CG (658.4 ± 167.2 c.c. and 629.8 ± 198.5 c.c., *P* = 0.35) before surgery, and tumor volumes were all < 50 c.c. at the primary end point after surgery (*P* = 0.17). After 2-year follow-up, one patient and two patients were died in the AG and CG, respectively. The mean OS sizes were 214.6 ± 145.7 c.c. and 467.2 ± 225.3 c.c. in AG and CG, respectively (*P* < 0.01). The results suggest that AOS intake reduces OS progression.

### AOS reduces the local OS recurrence

After 2-year follow-up, the rates of local OS recurrence were 44.9 and 68.7% in the AG and CG, respectively. The results suggest that long-term AOS intake reduces the rates of local OS recurrence.

### AOS affects biochemical characteristics of OS patients

Long-term AOS treatment improved the lipid profile in the patients from AG when compared with patients from CG. In AG, serum levels of TG, TC, LDL-C, and MDA were reduced, whereas the serum level of HDL-C was increased after 8 months; however, no statistically significant difference was observed between the two groups (*P* > 0.05, Table 2). Lipid pattern was improved further in AG after 16 months, and statistically significant difference was observed between the two groups (*P* < 0.05, Table 2). Similarly, the lipid pattern was improved significantly in AG after 24 months, and statistically significant difference was observed between the two groups (*P* < 0.05; Table 2). The results suggest that AOS significantly improved the lipid pattern in OS patients.

**Table 2:** Comparison of lipid pattern in osteosarcoma patients before and after therapy.

		**TG (mmol/L)**	**TC (mmol/L)**	**HDL-C (mmol/L)**	**LDL-C (mmol/L)**	**MDA (mmol/L)**
Before	AG	2.7 ± 1.1	5.7 ± 1.1	1.3 ± 0.2	3.8 ± 1.1	1.7 ± 0.2
	CG	2.8 ± 1.0	5.7 ± 1.3	1.2 ± 0.3	4.0 ± 1.0	1.6 ± 0.2
	*P*-value	0.72	0.82	0.65	0.72	0.85
8-month	AG	2.6 ± 1.2	5.5 ± 1.2	1.4 ± 0.2	3.5 ± 1.0	1.5 ± 0.3
	CG	2.7 ± 1.3	5.6 ± 1.1	1.3 ± 0.3	3.9 ± 1.0	1.7 ± 0.4
	*P*-value	0.32	0.21	0.17	0.23	0.26
16-month	AG	2.2 ± 1.2	5.1 ± 1.0	1.6 ± 0.3	3.2 ± 1.0	1.3 ± 0.2
	CG	2.8 ± 1.3	5.8 ± 1.1	1.3 ± 0.2	4.0 ± 1.3	1.8 ± 0.2
	*P*-value	0.02^*^	0.03^*^	0.04^*^	0.03^*^	0.02^*^
24-month	AG	1.8 ± 1.0	4.6 ± 1.3	1.7 ± 0.4	3.0 ± 1.0	0.9 ± 0.1
	CG	2.6 ± 1.2	5.4 ± 1.0	1.2 ± 0.2	3.9 ± 1.3	1.6 ± 0.2
	*P* value	0.01^*^	0.02^*^	0.01^*^	0.01^*^	0.01^*^

* *P < 0.05 via CG*.

As shown in Table 3, AOS increased the serum levels of SOD and GSH in AG when compared with the serum levels in CG (*P* < 0.05). By contrast, the levels of ALT were lower in AG than in CG (*P* < 0.05). Furthermore, long-term AOS treatment reduced the ratios of AST/ALT in AG when compared with CG (*P* < 0.05). The results suggest that long-term AOS treatment increases the antioxidant activities of OS patients.

**Table 3:** Biochemical parameters of enzyme activities.

		**SOD (U/ml)**	**GSH (ng/ml)**	**ALT (U/ml)**	**AST (U/ml)**	**AST/ALT**
Before	AG	25.4 ± 3.7	26.7 ± 4.9	46.8 ± 12.4	42.4 ± 15.6	0.9 ± 0.2
	CG	22.8 ± 3.2	25.6 ± 4.4	48.1 ± 11.8	44.8 ± 12.9	0.9 ± 0.2
	*P*-value	0.24	0.51	0.24	0.41	0.54
8-month	AG	26.8 ± 2.9	27.5 ± 4.7	45.4 ± 15.3	36.5 ± 11.3	0.8 ± 0.1
	CG	25.1 ± 3.4	29.6 ± 4.8	43.2 ± 16.1	38.8 ± 10.5	0.9 ± 0.2
	*P*-value	0.65	0.13	0.09	0.14	0.17
16-month	AG	25.4 ± 2.7	26.9 ± 4.9	43.4 ± 16.8	32.8 ± 9.6	0.7 ± 0.1
	CG	30.2 ± 3.4	35.8 ± 4.6	38.6 ± 17.5	40.3 ± 11.2	1.0 ± 0.2
	*P*-value	0.02^*^	0.03^*^	0.04^*^	0.01^*^	0.01^*^
24-month	AG	29.9 ± 2.3	32.1 ± 5.7	40.5 ± 18.9	28.0 ± 10.4	0.7 ± 0.1
	CG	46.9 ± 3.9	39.1 ± 4.6	30.8 ± 19. 6	41.5 ± 18.2	1.4 ± 0.2
	*P*-value	0.01^*^	0.02^*^	0.01^*^	0.01^*^	0.01^*^

* *P < 0.05 vs. CG*.

### AOS treatment reduces the serum levels of IL-1 beta and IL-6

**Figure 7:**
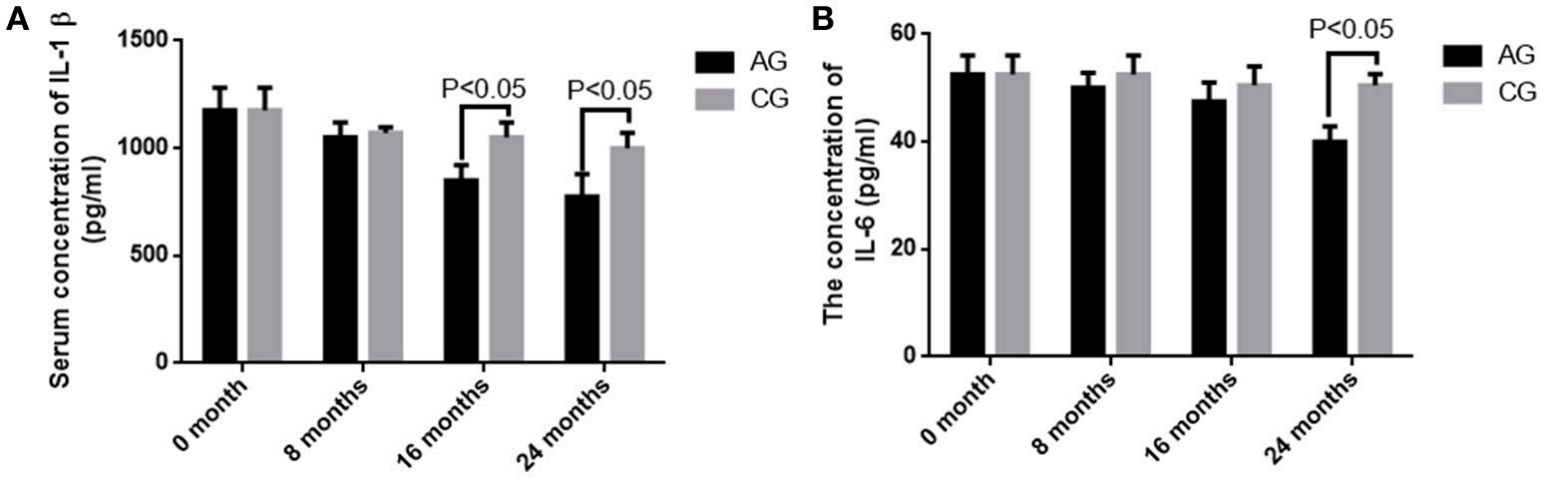
The concentrations of IL-1 beta and IL-6 in blood samples. **(A)** The serum level of IL-1 beta. **(B)** The serum level of IL-6. All data were presented as mean values ± *SD*. There are statistically significant differences if *P* < 0.05.

Before AOS treatment, no statistically significant difference was observed for serum levels of IL-1 beta and IL-6 between the two groups (*P* > 0.05). Comparatively, serum levels of IL-1 beta and IL-6 were decreased in both groups (*P* < 0.05), but no statistically significant difference was observed for the levels of IL-1 beta and IL-6 between the two groups after 8 months (*P* > 0.05). The levels of IL-1 beta and IL-6 were further reduced in both groups (*P* < 0.05), and statistically significant difference was observed for serum levels of IL-1 beta between the two groups after 16 months (Figure 7A, *P* < 0.05). Statistically significant difference was observed for serum levels of IL-6 between the two groups after 2 years (Figure 7B, *P* < 0.05).The results suggest that long-term AOS treatment greatly reduces the levels of IL-1 beta and IL-6. The serum levels of TNF were serially measured at 0, 8, 16, and 24 months. However, they were quite unstable in AG (range, 23–860 pg/mL) and CG (range, 17–920 pg/mL). Therefore, serum TNF levels were not correlated the occurrence of osteosarcoma.

## Discussion

OS is becoming more difficult to treat especially when it progresses. Thus, finding new ways to control OS progression is important. AOS shows significant inhibitory effects on osteosarcoma regrowth and local recurrence in OS patients after surgery. These results suggest that AOS has some beneficial effects in controlling OS development as a kind of natural products. High-molecular-weight AOS absorption is a common problem with osteosarcoma therapy. Low-molecular-weight AOS has no effects on OS, although it can be absorbed well. By contrast high-molecular weight AOS cannot be dissolved well in water, although it has effective therapeutic results on OS.

OS volume is an important character for predicting OS development and closely associated with OS progression (Figure 4). However, many factors cause OS, and its development may not be dependent on initial OS size. AOS treatment reduces OS volume in AG when compared with that in CG, suggesting that it can control OS better when subsequent treatment becomes difficult. OS recurrence also makes OS removal very difficult even with surgery. The pathogenesis of OS is associated with IL-1 beta (He et al., 2014) and IL-6 (Qi et al., 2016). Long-term intake of AOS reduces the levels of IL-1 beta and IL-6 (Figure 7), suggesting that it reduces OS recurrence of residual OS by affecting the levels of IL-1 beta and IL-6. According to an earlier report, enzymatically depolymerized unsaturated AOS induces TNF-α secretion, whereas the saturated AOS prepared by acid hydrolysis cannot affect TNF-α levels (Iwamoto et al., 2005). Thus, unsaturated AOS, made by alginate lyase, reduces the local OS recurrence by affecting TNF-α levels. By contrast, reverse results were also reported: TNF-α can promote osteosarcoma development by maintaining OS in an undifferentiated state (Mori et al., 2014). In the present work, serum levels of TNF-α were quite unstable in AG and CG groups. Serum TNF levels were not correlated with the occurrence of osteosarcoma. On the other hand, the induction of TNF-α by AOS also induces IL-1 and IL-6 according to a previous report (Iwamoto et al., 2005). However, in our work it was reduced (Figure 7). The difference may be due to the following reasons: (1) the previous work was performed in the mouse macrophage cell line RAW264.7, and cytokines were measured in human serum in our work; (2) the effects of AOS was only measured in < 4 days but in our work long-term (2 years) consumption of AOS was measured. To understand the difference better, further work is needed to confirm the results. Stimulation of innate immunity for cancer therapy has a long history. However, it may not be beneficial to reduce OS progression. Just as previously reported, high levels of TNF-α can promote osteosarcoma development by maintaining OS in an undifferentiated state (Mori et al., 2014). AOS reduces cytokines from high to normal levels and maintains the balance of innate immunity, which may be beneficial in reducing OS progression.

**Figure 8:**
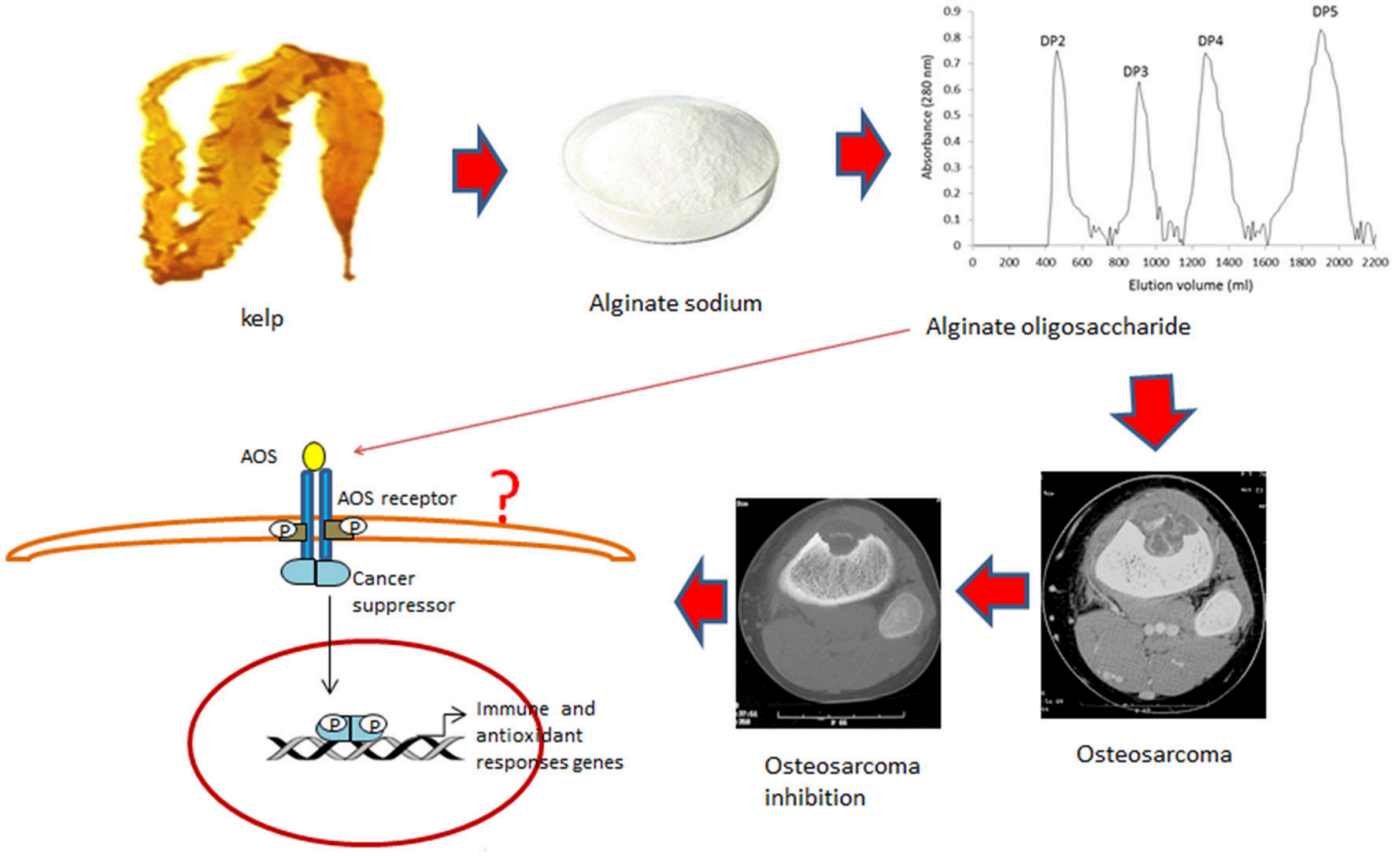
Cartoon illustrations of alginate oligosaccharide (AOS) inhibiting the growth of osteosarcoma and recurrence. AOS may bind the receptor on the membrane of osteosarcoma cells and activate cancer suppressors, which improve antioxidant and anti-inflammatory properties of OS patients.

Oxidative stress has a critical role in the progression of OS and promotes the invasiveness of osteosarcoma cells (Shin et al., 2016). AOS has potential antitumor applications (Fujihara and Nagumo, 1992) and reduces lipid peroxidation (Tusi et al., 2011). Present findings demonstrate that AOS shows obvious antioxidant properties for OS patients by improving the levels of SOD, and GSH and the ratio of AST/ALT (Table 3). However, the detailed molecular mechanisms of AOS function remain unclear. AOS may inhibit the growth of osteosarcoma and recurrence by activating some cancer suppressors, which have been reported to improve antioxidant and anti-inflammatory properties of patients (Figure 8; Hu et al., 2010).

AOS can be prepared from alginate by alginate lyase and available in most companies. Information on the side effects caused by AOS is limited. Alginate is extracted from fresh brown algae and simply purified by removing mitogenic and cytotoxic impurities because alginate polymers cannot be dissolved in solution. However, considering the potential mitogenic impurities responsible for the side effects of alginate, the women who developed osteosarcoma during pregnancy were excluded from the study.

The present study has some limitations: the OS population size still seems small to explore the possible mechanisms for OS treatment. Amputation has been widely considered for OS patients, and these patients were not enrolled in the present experiment because the operation will affect the study on OS recurrence. All patients took similar chemotherapy, so its effects on tumor size were not investigated. To confirm the present results, further work with a larger population is needed.

## Conclusions

AOS is a major component of alginate sodium and can reduce tumor size and OS recurrence. AOS DPs were prepared based on the enzymatic treatment. After the digestion of AOS, four main DPs were obtained from alginate by using the alginate lyase from *Agarivorans* sp. L11. Each DP was purified from DP mixture. Among these polymers, DP5 shows significant inhibitory functions on the growth of OS *in vitro*. This activity is lowered in a mixture of AOS with different DPs. DP5 can be produced by a few alginate lyase not by all the alginate lyases. Furthermore, DP5 has beneficial effects on OS patients: It can improve antioxidant properties of OS patients by increasing the serum levels of SOD and GSH, and reducing the serum levels of AST and ALT; It can improve lipid profile of OS patients by improving the serum levels of HDL-C and reducing the serum levels of TG, TC, LDL-C, and MDA; It can improve the anti-inflammatory properties of OS patients by reducing the serum levels of IL-1 beta and IL-6. Theoretically, specific oligosaccharides may have stronger inhibitory effects on OS progression. Therefore, future work is needed to identify the effects of purified DP5 of AOS on the OS progression and explore the possibility of alginate pentasaccharide as a potential antitumor drug for OS therapy.

## Author contributions

JC, YH, LZ, YJW, and SW recruited all osteosarcoma patients and designed the study. YZ, HG, DJ, and YTW performed all the experiment and analyzed the data. JC wrote the paper. All authors agreed to submit the work to present journal.

### Conflict of interest statement

The authors declare that the research was conducted in the absence of any commercial or financial relationships that could be construed as a potential conflict of interest.
